# Microbiome and resistome characterization of patients colonized with carbapenem-resistant Enterobacterales by long-read metagenomic next-generation sequencing of rectal swabs

**DOI:** 10.1093/jacamr/dlaf152

**Published:** 2025-08-28

**Authors:** John A Fissel, Yehudit Bergman, Victoria L Campodónico, Dana M Walsh, Brian Fanelli, Keshav Arogyaswamy, Jennie H Kwon, Aaron M Milstone, Pranita D Tamma, Patricia J Simner

**Affiliations:** TriCore Reference Laboratories, Albuquerque, NM, USA; Division of Medical Microbiology, Johns Hopkins University School of Medicine, Baltimore, MD, USA; Department of Pediatrics, Johns Hopkins University School of Medicine, Baltimore, MD, USA; Division of Medical Microbiology, Johns Hopkins University School of Medicine, Baltimore, MD, USA; Maryland Department of Health Laboratories Administration, Baltimore, MD, USA; Cmbio, Germantown, MD, USA; Cmbio, Germantown, MD, USA; Cmbio, Germantown, MD, USA; Division of Infectious Diseases, Washington University School of Medicine, St. Louis, MO, USA; Department of Pediatrics, Johns Hopkins University School of Medicine, Baltimore, MD, USA; Department of Pediatrics, Johns Hopkins University School of Medicine, Baltimore, MD, USA; Division of Medical Microbiology, Johns Hopkins University School of Medicine, Baltimore, MD, USA; Division of Infectious Diseases, Johns Hopkins University School of Medicine, Baltimore, MD, USA; Division of Clinical Microbiology, Department of Laboratory Medicine and Pathology, Mayo Clinic, Rochester, MN, USA

## Abstract

**Objectives:**

Evaluation of differences in the intestinal microbiome and resistome among high-risk patients (i.e. intensive care, oncology, transplant recipients) who are and are not colonized with carbapenem-resistant Enterobacterales (CRE).

**Methods:**

One hundred and twelve rectal swabs were obtained from 85 patients with known CRE colonization status and cohorted. Long-read metagenomic next-generation sequencing (mNGS) was performed on rectal swabs. Microbiome and resistome analysis were performed by assessing α-diversity, β-diversity, relative abundance assessment and linear discriminant analysis effect size (LEfSe), comparing patients colonized (CRE positive) and not colonized (CRE negative) with CRE. Longitudinal analysis of sequential swabs collected over multiple hospital encounters on a subset of patients was performed at the patient level.

**Results:**

The microbiomes of cohorts were similar when comparing α- and β-diversity measures and relative abundance. LEfSe analysis identified Gram-negative pathobionts enriched among CRE-positive samples and Gram-positive taxa enriched among CRE-negative samples. α-Diversity of the resistome differed at class, gene and allele levels. Relative abundance and LEfSe analysis demonstrated enrichment of genes conferring β-lactam resistance among CRE-positive patients; LEfSe also demonstrated enrichment of antimicrobial resistance genes to multiple antimicrobial classes. At the patient level, fluctuations in the microbiome and resistome among longitudinally collected swabs were associated with antibiotic exposure.

**Conclusions:**

Differences between the microbiomes of CRE-positive- and CRE-negative-colonized patients at the cohort level were relatively muted, whereas statistically significant differences were observed among their resistomes. In patients followed longitudinally, shifts in microbiome and resistome composition were dramatic in between encounters and antibiotic exposures.

## Introduction

Carbapenem-resistant Enterobacterales (CRE) present an ongoing public health threat.^1^ Treatment options for CRE infections are limited and outcomes are worse for MDR organism infections. Therefore, identifying microbial factors that decrease the risk of CRE colonization and reduce the likelihood of subsequent progression to CRE infection are important to thwart challenging future treatment dilemmas. Metagenomic next-generation sequencing (mNGS) has become an increasingly useful tool to investigate the gastrointestinal microbiome and resistome. Opportunities exist to leverage this technology for clinical use by identifying characteristic communities and antimicrobial resistance (AMR) gene repertoires that make the human intestinal tract more hospitable to CRE colonization.^2,3^

Several studies have highlighted the utility of Oxford Nanopore long-read sequencing to characterize the microbiome and resistome of human stool specimens. One study using mNGS interrogated the microbiomes and resistomes of stool samples from healthy and ill pre-term infants to determine longitudinal microbiota profiles, identify gut-associated pathogens and characterize AMR profiles.^2^ Another study demonstrated comparable results to short-read Illumina sequencing for surveillance of AMR organisms.^4^ Moreover, a study using 16S rRNA sequencing with Illumina MiSeq compared the microbiomes of stool specimens collected from hospitalized non-CRE carriers and CRE carriers. CRE-colonized patients had reduced diversity and dysbiotic microbiota enriched with Enterobacterales.^5^

Obtaining rectal swabs is arguably more logistically feasible than obtaining stool specimens from hospitalized patients. Rectal swabs have broad correlation and adequate capture of patient microbiome signatures compared with stool specimens.^6,7^ As part of larger surveillance efforts at The Johns Hopkins Hospital, more than 4000 high-risk patients (i.e. ICU, oncology, solid organ and HSCT recipients) were screened for CRE intestinal colonization at admission and weekly thereafter by culturing over 10 000 rectal swabs.^8–10^ In the present study, a convenience set of these surveillance samples was further investigated to characterize differences in diversity in the microbiomes and resistomes of patients colonized or not colonized with CRE. Furthermore, we present longitudinal microbiome and resistome data for two patients who acquired CRE during hospitalization and progressed to infection, and a third patient who was initially CRE negative then became subsequently colonized with CRE.

## Materials and methods

### Cohort selection

For cohort-level analysis, mNGS was performed on a convenience set of 92 rectal swabs from 85 patients that were collected between January 2016 and June 2017. Patients were categorized into five groups: (1) patients negative for CRE at hospital admission (*n* = 27); (2) patients negative for CRE at hospital admission but who acquired CRE during the hospitalization (*n* = 19); (3) patients negative for CRE at admission but who later acquired CRE during the hospitalization and progressed to CRE infection (*n* = 2); (4) patients positive for CRE at admission but who did not progress to CRE infection (*n* = 35); and (5) patients positive for CRE at admission and who progressed to CRE infection (*n* = 2). The convenience set was enriched for CRE-positive samples in patient groups 2–5 for which multiple swabs were collected per patient. Patients who were positive for CRE at any point were grouped together as one cohort (*n* = 58 patients; CRE positive); their microbiomes and resistomes were compared against the cohort negative for CRE (*n* = 27 patients, CRE negative). Seven patients who had multiple swabs sequenced had only their initial specimen included in the cohort-level analysis. These seven patients included both patients who were CRE negative at admission but acquired CRE during admission and progressed to infection, along with another five patients who were CRE negative at admission but acquired CRE during hospitalization.

### Patients selected for longitudinal analysis

For longitudinal analysis, 26 swabs were evaluated from three patients. The two patients who were negative for CRE at admission but later acquired CRE and progressed to infection [Patient 2 (12 swabs over three admissions in 6 months] and Patient 22 (5 swabs over three admissions in 2 months)] and the patient who acquired CRE but did not progress to infection (Patient 47 with 9 swabs over two admissions in 4 months) were followed longitudinally; changes in their microbiome and resistome were observed over time. Initial swabs for the longitudinal patients were included in the cohort analysis.

### Sample collection and standard-of-care methodology

Samples were collected as part of a larger surveillance study at The Johns Hopkins Hospital. Rectal swabs were collected on admission and weekly thereafter using ESwabs.^8–10^ Carbapenem-resistant organisms were selected using the direct MacConkey plate method, as previously described.^9^ CRE identified were further characterized by the modified carbapenem inactivation method to detect carbapenemase production; if positive, WGS was performed as previously described.^11,12^

### Nucleic acid extraction

Genomic DNA was extracted from rectal swab Amies broth using the DNeasy PowerSoil Pro Kit (QIAGEN, Hilden, Germany). DNA was sheared using Covaris G-tubes to 10 kb at 5000 rpm for 1 min on each side. Long-read genomic sequencing was performed using the GridION X5 (Oxford, UK) sequencing instrument. Each Nanopore sequencing library was prepared using up to 5 µg of DNA with the 1D ligation kit (SQK-LSK108, Oxford Nanopore Technologies) and sequenced using R9.4.1 flow cells (FLO-MIN106). Five samples were run per flow cell. MinKNOW software was used to collect sequencing data using high-accuracy basecalling (HAC) and Guppy 5.0.11. The median number of reads per specimen was 543 019, the median reads mapped to bacteria was 110 455, and the median reads mapped to AMR genes was 4184. Additional sequencing run metrics are summarized in the Supplementary material (available as Supplementary data at *JAC-AMR* Online). The sequencing data underwent removal of host reads using minimap2 for alignment to the CHM13 human genome (T2T-CHM13v2) and SAMtools for extraction of non-aligned sequences.^13,14^ The non-human reads were deposited to the Sequence Read Archive (SRA) under BioProject 1088077.

### Analysis via CosmosID-HUB microbiome platform

Unassembled sequencing reads were directly analysed by the CosmosID-HUB Microbiome Platform (CosmosID Inc., Germantown, MD, USA), as described elsewhere, for multi-kingdom microbiome analysis, profiling of antibiotic resistance and virulence genes, and quantification of organism relative abundance.^15–18^ Briefly, the system utilizes curated genome databases and a high-performance data-mining algorithm that rapidly disambiguates hundreds of millions of metagenomic sequence reads into discrete microorganisms engendering specific sequences. AMR genes (i.e. the resistome) in the microbiome are identified by querying unassembled sequence reads against the CosmosID curated antibiotic resistance database that was established using the Antibiotic Resistance Genes Database (ARDB) and Antibiotic Resistance Gene ANNOTation (ARG-ANNOT).^19,20^ The AMR gene phylogeny included class (antimicrobial agent class), gene (AMR gene name) and type (AMR gene allele). For AMR coverage, a 100% identity threshold with five unique kmers (kmer length: 30 bp) was required for identification down to the lowest level possible (e.g. AMR allele). Further details on CosmosID bioinformatic algorithms can be found on their website (https://docs.cosmosid.com/docs/kepler-microbiome-profiler#pre-computational-stage-for-curating-and-building-a-comprehensive-biomarker-database-genbook).

### Linear discriminant analysis effect size (LEfSe)

LEfSe figures were generated using the LEfSe tool from the Huttenhower lab, based on bacterial and AMR relative abundance matrices from CosmosID taxonomic analysis.^21^ LEfSe is calculated with a Kruskal–Wallis α value of 0.05, a Wilcoxon α value of 0.05, and a logarithmic linear discriminant analysis (LDA) score threshold of 2.0. In the LEfSe figures, an LDA score of 2.0 was considered significant.

### Relative abundance stacked bars

Stacked bar figures were generated from bacterial and AMR relative abundance matrices from CosmosID taxonomic analysis. Stacked bar figures for each group were generated using the R package ggplot2.

### α-Diversity boxplots (with Wilcoxon rank-sum test)

α-Diversity boxplots were calculated from the species-level abundance score matrices from CosmosID taxonomic analysis. Chao, Simpson and Shannon α-diversity metrics were calculated in R using the R package Vegan. Wilcoxon rank-sum tests were performed between groups using the R package ggsignif. Boxplots with overlaid significance in *P* value format were generated using the R package ggplot2.

### β-Diversity principal coordinate analysis (PCoA) with PERMANOVA

β-Diversity PCoA was performed from bacterial and AMR relative abundance matrices from CosmosID taxonomic analysis. Bray–Curtis and Jaccard diversities were calculated in R using the R package Vegan with the function vegdist, and PCoA tables were generated using Vegan’s function pcoa. PERMANOVA tests for each distance matrix were generated using Vegan’s function adonis2. Plots were visualized using the R package ggpubr.

### Ethics

This study was approved by the Johns Hopkins University institutional review board, with a waiver of informed consent.

## Results

### Cohort-level microbiome and resistome characterization

**Figure 1.**
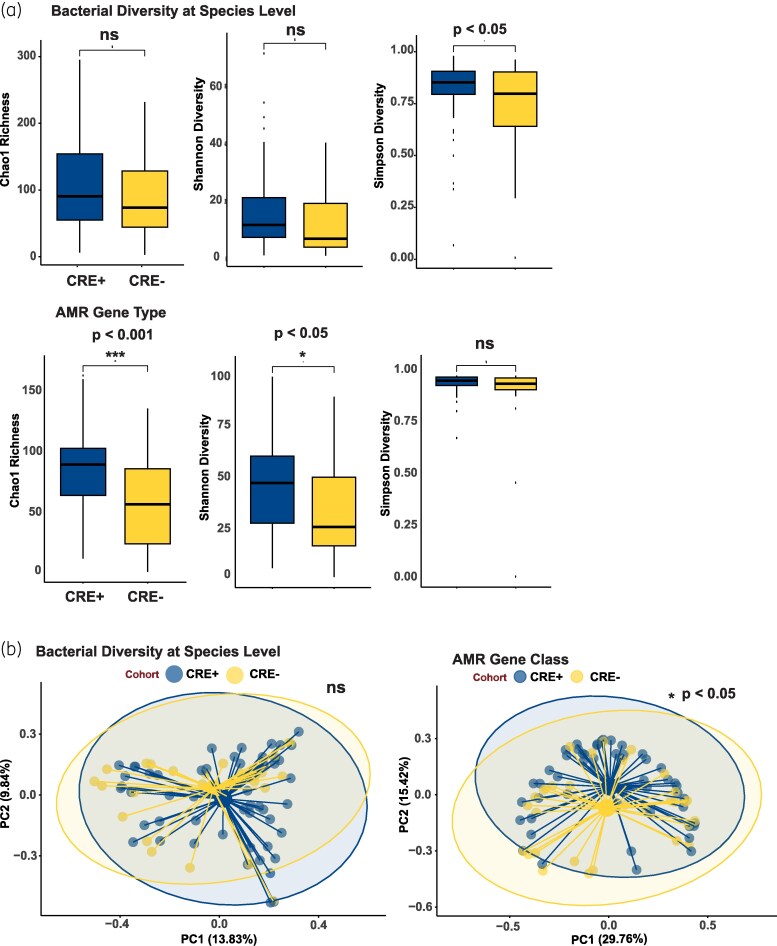
α-Diversity of cohort microbiome and resistome measured by Chao richness index, Shannon diversity index and Simpson diversity index at the bacterial species level and AMR gene-type level (*P* < 0.05) (a). β-Diversity PCoA of the microbiome at the bacterial species level and of the resistome at the AMR gene-class level by Bray–Curtis analysis (*P* < 0.05) (b).

α-Diversity of the microbiomes and resistomes of CRE-positive and -negative patient cohorts were compared. There were no observed differences in microbiome diversity at the phylum and genus (data not shown) levels, but at the species level the CRE-positive cohort had a higher Simpson diversity index (*P* < 0.05), likely reflecting a high level of dysbiosis in this population regardless of CRE colonization status (Figure 1a). Furthermore, there were no statistically significant differences between the species total count between CRE-positive versus CRE-negative cohorts (*P* = 0.38). However, CRE-positive patients had increased AMR diversity at the class and gene level by Chao richness estimate (*P* < 0.01) but not by Shannon and Simpson diversity indices (data not shown). An increase in α-diversity for AMR gene types was observed among CRE-positive patients by Chao richness estimate and Shannon diversity index, but not by Simpson diversity index (Figure 1a). Furthermore, a statistically significant difference between AMR gene count was observed between CRE-positive and -negative cohorts (*P* = 0.007).

To further characterize the overall composition of microbiomes and resistomes of patients, Bray–Curtis PCoA was applied. Microbiomes of patients who remained CRE negative and CRE-positive patients were similar at the phylum, genus (data not shown) and species level (Figure 1b). The resistomes of the two groups differed at the highest level of AMR taxonomy, the AMR class level (Figure 1b), but were similar at the lower taxonomic levels, the AMR-gene and gene-type levels (data not shown).

**Figure 2.**
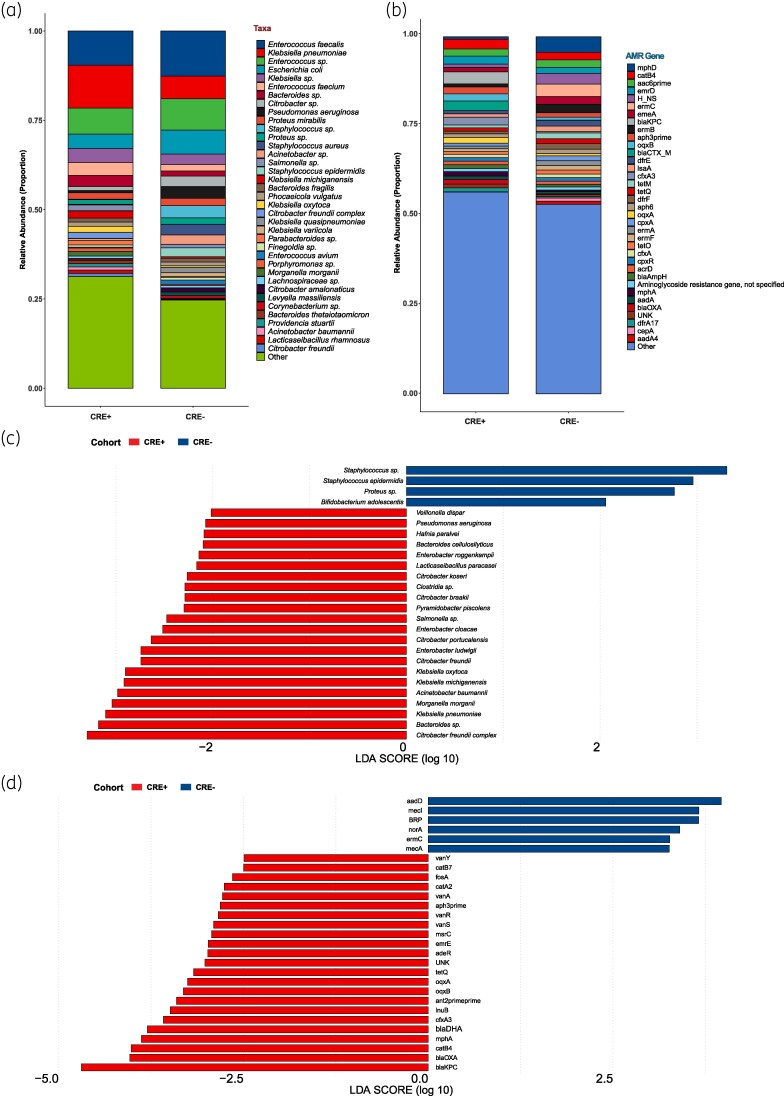
Relative abundances of cohort microbiomes at the species level (a) and resistomes at the AMR gene level (b). LEfSe analysis of cohort microbiome at the species level (c) and resistome at the AMR gene level (d). Blue bars to the right convey that the organism or resistance gene in that group is more abundant in the CRE-negative cohort. Red bars to the left convey that the organism or resistance gene is more abundant in the CRE-positive group.

The relative abundances of organisms and AMR classes/genes present in the microbiome were also assessed. The relative abundances of the individual taxa for each cohort at any taxonomic rank were similar, as assessed by the Wilcoxon rank-sum test (Figure 2a). As expected, at the AMR-class level there was an increase in the abundance of genes that confer β-lactam resistance in patients who were CRE positive (*P*-adjusted Holm = 0.003; Figure 2b).

Unlike α- and β-diversity measures and relative abundance analyses, LEfSe yielded significant enrichments in the microbiomes of the two cohorts (Figure 2c). Expectedly, Enterobacterales were enriched in the CRE-positive cohort, as were *Acinetobacter baumannii*, *Bacteroides* species and *Pseudomonas aeruginosa*. Conversely, the microbiomes of CRE-negative patients were enriched with Gram-positive taxa, including *Staphylococcus* species. In both CRE-positive and -negative cohorts, there were enrichments for organisms typically considered commensals such as *Bacteroides* spp. (CRE positive) and *Bifidobacterium adolescentis* (CRE negative), respectively.

Corroborating the relative abundance analysis finding for the resistome, LEfSe at the class level indicated that genes that confer β-lactam resistance were enriched in CRE-positive patients (Figure 2d). At the gene level, LEfSe indicated there was enrichment of the carbapenemase gene, *bla*_KPC_, the most prevalent carbapenemase found among the CRE-positive cohort. In addition, there was enrichment of other β-lactamases conferring β-lactam resistance (*bla*_OXA_, *bla*_CTX-M_, *bla*_DHA_, *cfxA3*), macrolide resistance [*mph*(A), *msr*(C)], aminoglycoside resistance [*ant(2′′)*, *aph(3′)*], fluoroquinolone resistance (*ogxA*, *oqxB*), chloramphenicol resistance (*catB4*, *catB7*), tetracycline resistance [*tet*(Q)], fosfomycin resistance (*fosA*) and glycopeptide resistance (*vanA*, *vanY*, *vanS*, *vanR*). There was also increased abundance of genes associated with efflux pumps (*emrE*, *adeR*) and porins (*oprJ*, *oprM*, *oprD*, *triABC*, *opmH*, *opmE*) conferring resistance to multiple drugs. The enriched AMR genes by LEfSe in the CRE-positive cohort are consistent with the enriched organisms identified in the microbiome. For example, many of the efflux pumps and porins that were enriched were associated with *P. aeruginosa* and *A. baumannii*. The one exception is enrichment of the vancomycin resistance operon in the absence of significant enrichment of enterococci.

Among the CRE-negative cohort, genes conferring resistance to aminoglycosides (*aadD*), bleomycin (*ble*MBL), fluoroquinolones (*norA*), methicillin (*mecA*) and macrolides [*erm*(C)] were enriched by LEfSe analysis. There was also an increased abundance of *mecI*, a regulatory gene for *mec*A expression. The enrichment of *mecA* and associated regulatory genes, among other AMR genes, is consistent with the enrichment of *Staphylococcus* species by LEfSe.

### Longitudinal evaluation of the microbiome and resistome of individual patients

**Figure 3.**
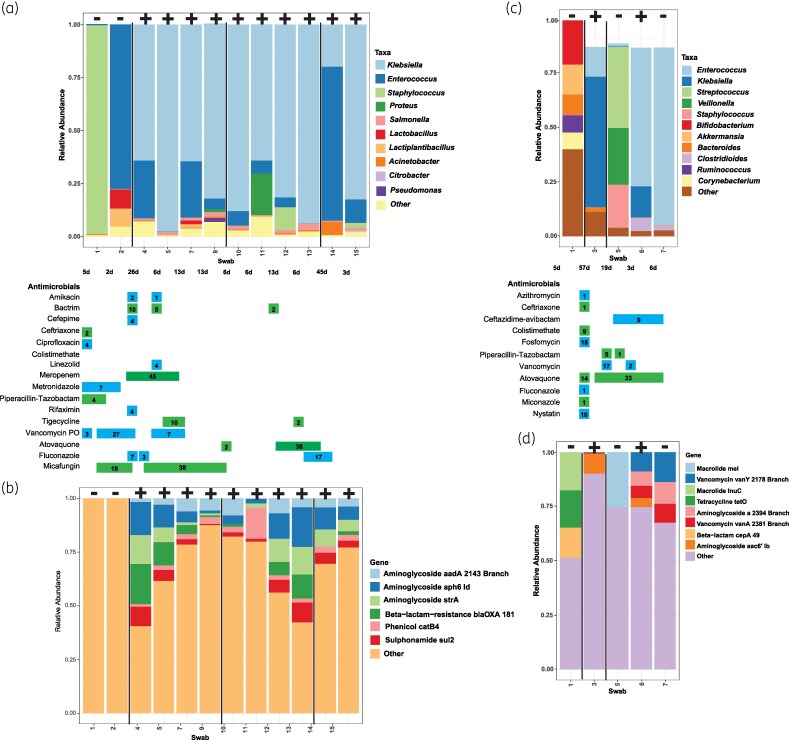
Relative abundance of bacterial taxa (a and c) and AMR genes (b and d) for Patients 2 (a and b) and 22 (c and d) of our cohort who acquired CRE and progressed to infection. Vertical black bars represent different patient encounters (a and b) and each swab is designated as being positive (+) or negative (−) for CRE by standard-of-care testing at the top of the figure. For Patients 2 and 22, a carbapenem-resistant *K. pneumoniae* was recovered by rectal surveillance and found to harbour the following antimicrobial resistance genes: (a and b) *bla*_NDM-1_, *bla*_OXA-181-like_, *bla*_CTX-M-15_, *bla*_CMY-4_, *bla*_SHV-1_ and *bla*_TEM-1B_; and (c and d) *bla*_KPC-2_, *bla*_LEN12_ and *bla*_OXA-9_ . Patient antimicrobial therapy histories are displayed with the number of days on therapy indicated by numbers in bars, and days between swab collections indicated below the *x*-axis stacked bar plot.

Patients with multiple datapoints had shifts in relative abundances in their microbiomes and resistomes after incident colonization or progression to infection. Patient 2, who had a history of ulcerative colitis and cirrhosis secondary to autoimmune hepatitis, received a liver transplant 17 days after admission and was followed over the course of 6 months. The patient initially had negative CRE surveillance cultures, then became colonized with an NDM and OXA-181-producing *Klebsiella pneumoniae*, developed bacteraemia with the organism 6 days after transplant, and remained CRE colonized for the duration of their hospital stay (Figure 3a and b). At the time of diagnosis with CRE infection, the patient was on Day 17 of meropenem in addition to other anti-infectives (i.e. Day 18 micafungin, Day 6 trimethoprim/sulfamethoxazole, Day 19 oral vancomycin). At the species level, the microbiome showed a dramatic shift following admission. Initially, the microbiome was transiently dominated by Gram-positive taxa (*Staphylococcus*, *Enterococcus* and *Lactobacillus* spp.) while being CRE negative. Following CRE colonization, *Klebsiella* spp. became the dominant taxon. This dominance was also observed in surveillance cultures as *K. pneumoniae* was recovered from multiple weekly cultures after the observed shift in the microbiome (Figure 3a). Molecular characterization of the recovered *K. pneumoniae* from rectal surveillance cultures revealed that the isolate harboured *bla*_TEM-1B_, *bla*_SHV-1_, *bla*_CTX-M-15_, *bla*_CMY-4_, *bla*_NDM-1_ and *bla*_OXA-181._ This was reflected in the resistome analysis as genes conferring β-lactam resistance became more abundant compared with prior to CRE acquisition (Figure 3b). *Klebsiella* spp. were consistently isolated from rectal surveillance culture and were present in high abundance by mNGS analysis of the rectal swabs, while *bla*_OXA-181_ abundance was at its highest abundance during a 45 day treatment duration of meropenem. Once meropenem was discontinued, the abundance of *bla*_OXA-181_ decreased over the course of 6 weeks.

Patient 22 had microscopic polyangiitis and was receiving chronic prednisone therapy. The patient’s microbiome fluctuated over a 2 month duration (Figure 3c and d). Patient 22 was also initially CRE negative on surveillance cultures then became colonized with a KPC-producing *K. pneumoniae* on their second hospital admission, when they progressed to a *K. pneumoniae* bloodstream infection. The patient completed 14 days of oral fosfomycin treatment the day prior to the CRE infection. Initially, the patient had a diverse community consisting of mostly commensal organisms such as *Bifidobacterium*, *Akkermansia* and diverse genera in lower relative abundance, represented by ‘other’ (Figure 3c and d). However, following the first patient encounter, the microbiome shifted dramatically towards *Klebsiella* spp. for the next encounter, then *Streptococcus* and *Enterococcus* after exposure to various antimicrobials, including ceftriaxone, piperacillin/tazobactam and vancomycin. The resistome analysis did not highlight the enrichment of β-lactamase genes associated with the KPC-producing *K. pneumoniae* (Figure 3d). However, it demonstrated the enrichment of vancomycin resistance genes associated with VRE subsequent to vancomycin exposure.

**Figure 4.**
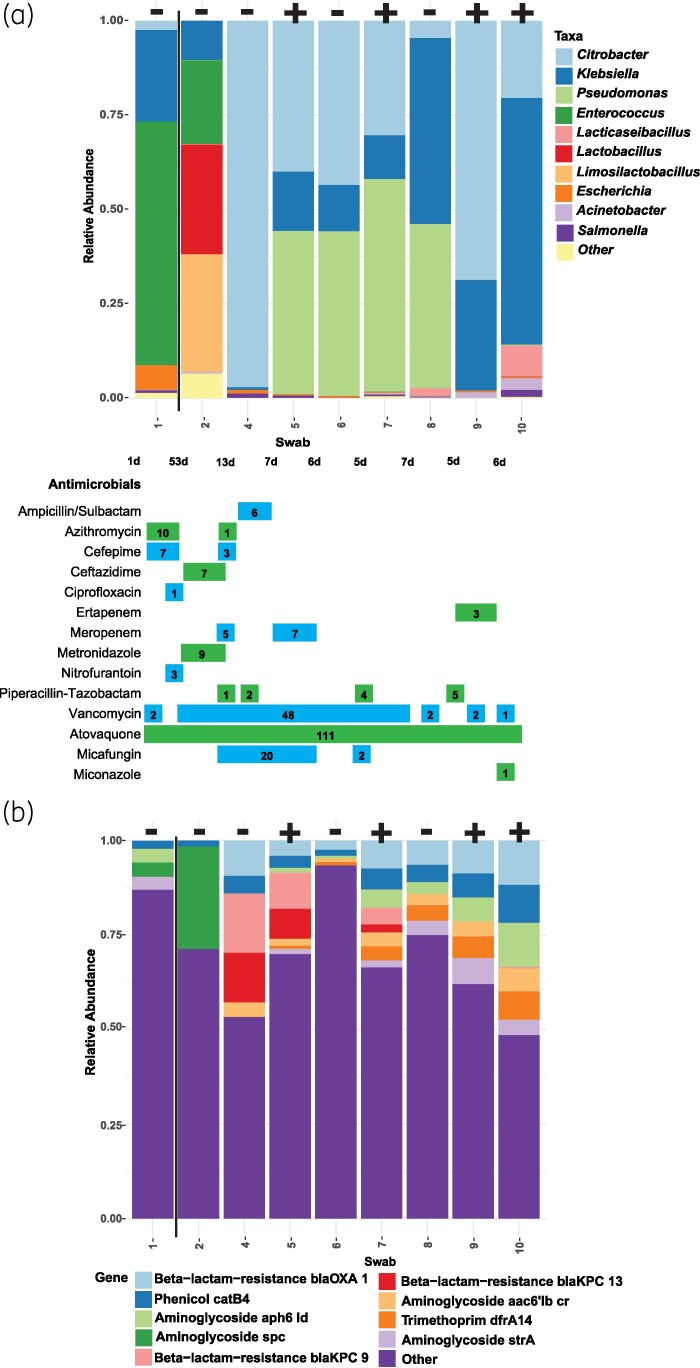
Relative abundances of bacterial taxa (a) and AMR genes (b) for Patient 47 of our cohort who was initially negative then acquired CRE. Each swab is designated as being positive or negative for CRE by standard-of-care testing. No AMR genes were detected by metagenomic analysis for Swab 3 so it is omitted from (b). CRE detected by standard of care were KPC, TEM-WT, NDM, CTX-M-15 and SHV-WT producers. Patient antimicrobial therapy histories are displayed with the number of days on therapy indicated by numbers in bars, and days between swab collections indicated below the *x*-axis stacked bar plot.

Patient 47 had a history of polymyositis, interstitial lung disease and Crohn’s disease and was receiving chronic treatment with prednisone. This patient was initially CRE negative, then became colonized with a KPC-producing *Citrobacter amalonaticus*, but did not progress to infection. At the time CRE was detected, the patient had been antibiotic-free for 5 days (Figure 4a). However, they had received extensive antimicrobial therapy within the prior 2 months (i.e. 7 days meropenem, 6 days piperacillin/tazobactam, 21 days micafungin, 21 days IV vancomycin, 49 days oral vancomycin). Initially the microbiome was predominantly composed of *Enterococcus*, then in the next encounter shifted towards *Lactobacillus, Limosilactobacillus* and *Lacticaseibacillus* 53 days later. Another shift occurred 13 days later and *Citrobacter*, *Pseudomonas and Klebsiella* spp. dominated the microbiome over the remaining encounter. The resistome showed an increase in genes conferring β-lactam resistance and aminoglycoside resistance (Figure 4b). Characterization of the *C. amalonaticus* recovered in rectal surveillance cultures revealed the isolate harboured *bla*_KPC_, *bla*_CTX-M-15_, *bla*_OXA-1_, *bla*_SHV-28_ and *bla*_TEM-1B_. Resistome analysis reflected the enrichment of AMR genes harboured by the *C. amalonaticus* isolate when present in increased abundance in the microbiome.

## Discussion

Despite significant changes in microbiome and resistome composition over time in our longitudinal patients, we did not observe large differences in diversity or relative abundance between patients colonized with or without CRE at the cohort level, especially at the microbiome level. The cohorts did show a difference in β-diversity at the AMR gene-class level, likely due to the representation of AMR mechanisms associated with Gram-negative and Gram-positive organisms seen in the CRE-positive and CRE-negative cohorts, respectively. A possible explanation for the muted differences by other analyses and levels of taxonomy is that all of the included patients were either critically ill or severely immunocompromised and exposed to often prolonged durations of broad-spectrum antimicrobial therapy among other agents likely to impact intestinal flora (e.g. chemotherapeutic agents, corticosteroids) perturbing their normal microbiota.^22^ To this point, we observed among the longitudinal sampling of patients where fluctuations in the microbiome and resistomes were linked to antimicrobials the patients were receiving. For example, increased abundance of CRE after prolonged meropenem exposure.

Interestingly, applying LEfSe assessment at the cohort level, the microbiomes of CRE-negative patients were enriched for Gram-positive organisms, while microbiomes of CRE-positive patients were enriched for Gram-negative organisms. Enterobacterales make up the majority of the enrichment for Gram-negative organisms but other Gram-negative organisms were also present, including non-fermenting organisms such as *A. baumannii* and *P. aeruginosa*. These organisms have multiple complex intrinsic mechanisms of resistance, also enriched by resistome analysis; under shifting antimicrobial selective pressure, perhaps these organisms are well suited to persist. Furthermore, resistome assessment correlated with the organisms enriched by LEfSe. Consistent with the CRE colonization status, Enterobacterales and β-lactamase genes, in particular *bla*_KPC_, were enriched among CRE-positive specimens. For CRE-negative specimens, *Staphylococcus* species were enriched along with *mecA*, which encodes methicillin resistance among staphylococci. Other studies have shown that anaerobic organisms may prevent colonization and proliferation of resistant pathogens.^23–25^ However, while we observed that *B. adolescentis* was enriched in CRE-negative patients, we found that another anaerobe (*Bacteroides* sp.) was enriched in CRE-positive patients, perhaps owing to possible broad dysbiosis in this high-risk patient population. Alternatively, *Bacteroides* species are known to be the most commonly isolated antimicrobial-resistant anaerobes and it is possible that AMR genes harboured by this organism may contribute to selection among the CRE-positive cohort (e.g. *cfxA3* enriched in CRE positive specimens in this study).^26,27^ Other studies show oxygen levels decrease proceeding from mucosa to lumen, suggesting that stool specimens tend to be more enriched for anaerobic bacteria compared with rectal swab and mucosal tissue specimens, raising the possibility that perhaps the choice of specimen may play a role in anaerobic organism recovery.^6,7,28–30^

This study has limitations. For cohort analysis it could be possible that antibiotic therapy in both CRE-negative and CRE-positive groups could have shifted diversity and composition in similar ways, reducing any observed differences that could be detected. In addition, we did not link patient-level characteristics to the microbiome and resistome data at a cohort level. Experimental controls were not included while performing metagenomic analysis. Finally, we cannot confirm that rectal swabs were a sufficient proxy for a traditional stool specimen. Given the increased abundance of Gram-positive organisms such as *Staphylococcus* species, here may also be differences in the quality of specimens collected for each cohort. The presence of these organisms could be an indication of skin colonization due to a more superficial swabbing technique.

Overall, differences between the CRE-positive and CRE-negative patients at the cohort level were relatively muted and may reflect the limitations of using cohort-level research approaches to study shifts in the microbiome and resistome of critically ill patients. However, there was significant enrichment of bacterial taxa and genes conferring β-lactam resistance in the CRE-positive cohort. In patients who were followed longitudinally, the shifts in microbiome and resistome composition were dramatic, and often reflective of recent antibiotic exposure. During the time that these patients were followed, they never returned to a composition similar to their baseline. Similar findings were observed among healthy adults exposed to antibiotics, leading to acute and persistent shifts in the microbiome and resistome.^28^ The use of multiple timepoints for individual patients along with antimicrobial therapy history, provide useful context as the microbiome and resistome shifts over time. A single timepoint analysis may provide insight into the microbiome and resistome of critically ill patients. However, a longitudinal approach may provide stronger insights into the dynamic shifts in the microbiome and resistome that occur over time in response to antimicrobial therapy, among other microbial disruption factors.

## Supplementary Material

dlaf152_Supplementary_Data
